# Evaluation of extraction methods for co-isolation of nucleic acid from human saliva for forensic body fluid identification

**DOI:** 10.1007/s00414-025-03641-9

**Published:** 2025-11-07

**Authors:** Minakshi Singh, Claire V.S. Pike, S. Krithika, Timothy J. Hearn

**Affiliations:** 1 Faculty of Science and Engineering, Anglia Ruskin University, Cambridge, CB1 1PT United Kingdom; 2 Department of Genomic Medicine, School of Clinical Medicine, University of Cambridge, Cambridge, CB2 0QQ United Kingdom

**Keywords:** Forensic science, Forensic genetics, Body fluid identification (BFID), DNA/RNA, MicroRNA, Saliva

## Abstract

MicroRNAs (miRNAs) have emerged as promising biomarkers for the identification of forensically significant body fluids, aiding crime scene reconstruction and the identification of biological material donors. However, despite their potential, inter-study discrepancies and lack of reproducibility have limited their forensic application, particularly in body fluid identification. A major challenge lies in the variability introduced by different nucleic acid extraction methods. This study aimed to evaluate the co-extraction performance and miRNA detection efficiency of three commonly used nucleic acid extraction kits (AccuPrep Genomic DNA Extraction Kit, QIAamp DNA Mini Kit, and miRNeasy Tissue/Cells Advanced Micro Kit) across five saliva input volumes (400 µL, 200 µL, 100 µL, 50 µL, and 25 µL). To assess yield, purity, robustness (CV%), and sensitivity, two quantification platforms were used, followed by a two-step reverse transcription quantitative polymerase chain reaction (RT-qPCR) protocol to evaluate the impact of extraction methods on miRNA expression. Our results demonstrate that both the extraction procedure and the quantity of input material significantly influences nucleic acid recovery. Additionally, we observe variations in miRNA levels depending on the extraction method used. Surprisingly, the kit designed specifically for miRNA extraction yielded relatively poor miRNA recovery. In contrast, the DNA extraction kit (AccuPrep Genomic DNA Extraction Kit) produced the highest nucleic acid yield with moderate purity and showed the lowest Cq values for miRNA targets, indicating better miRNA detection. These findings underscore the importance of the choice of extraction kit, as it can significantly influence both the yield and quality of nucleic acids detected, and the extension, accuracy and reliability of miRNA-based forensic analyses.

**Supplementary Information:**

The online version contains supplementary material available at 10.1007/s00414-025-03641-9.

## Introduction

In forensic science, the term “body fluid” refers to “suspect stain collected from a surface outside of the originating body” [1]. Human body fluids such as blood, saliva, semen, vaginal secretions, and menstrual blood are frequently encountered at crime scenes, as well as on suspects, victims, and evidentiary objects. These fluids are valuable sources of DNA, facilitating the identification of the biological material’s donor through DNA profiling. Additionally, the specific type of body fluid can provide crucial insights into the activities surrounding the commission of a crime [2–4], helping forensic investigators to establish connections between the victim, suspect, and crime scene. This is particularly important in sexual assault cases, where identifying the source of the biological fluid, along with obtaining a DNA profile, is essential for linking the origin of the fluid to the criminal act rather than to incidental contact [5, 6].

Traditionally, body fluid identification (BFID) relied on various serological tests, including enzymatic, immunological, chemical, spectroscopic, and microscopic methods [7, 8]. These tests, which can be either presumptive or confirmatory, have several limitations, including limited sensitivity, low specificity, high sample consumption, and the potential for sample destruction. Moreover, these procedures are often time-consuming, labour-intensive, and may involve reagents that are unstable over time. These constraints highlight the need for more advanced, reliable, and efficient methods for BFID in forensic science [7, 9].

Recent advances in forensic genetics have introduced novel molecular biomarkers such as DNA methylation, mRNA, and miRNA for body fluid identification (BFID) [8, 10]. Both mRNA and DNA methylation markers present specific challenges for forensic BFID. While mRNA molecules are highly susceptible to degradation by ribonucleases and environmental conditions, posing challenges for consistent detection, particularly in aged or environmentally exposed forensic samples [11], DNA methylation patterns can be influenced by individual-specific factors such as environmental exposures and aging, reducing their suitability for large-scale forensic BFID applications [12, 13].

MicroRNAs (miRNAs) are small, non-coding, single-stranded RNA molecules, typically 18–24 nucleotides in length, that regulate gene expression at the post-transcriptional level [14–16]. These molecules are present in plants, animals, and viruses, and they exhibit tissue-specific expression [17]. Moreover, miRNAs are found in various extracellular bodily fluids. Due to their high tissue specificity, abundance, and remarkable stability, miRNAs have gained significant interest in forensic science [18–21]. They are extensively studied in forensic casework and are increasingly proposed as novel biomarkers for BFID [22–42].

**Fig. 1:**
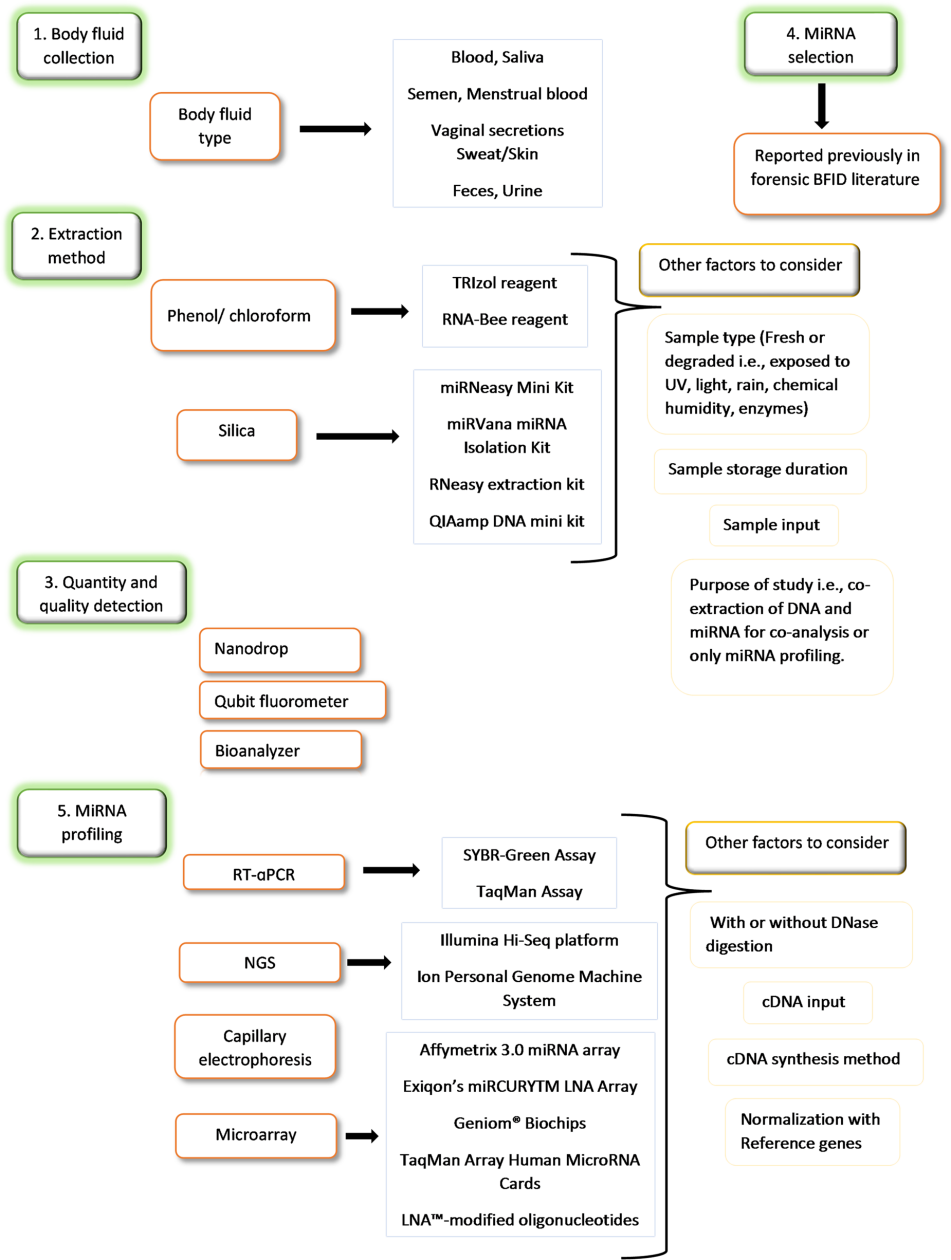
A multi-step workflow of miRNA profiling for identification of forensically significant body fluid. One can either first collect body fluid (1) or can select miRNA markers for analysis (4) Then, extraction is performed using a commercially available isolation kit (2) followed by quality and quantity assessment of nucleic acid (3) For extraction method selection factors to consider include sample type, storage and input as well as the purpose of the study. Finally, miRNA analysis is done using one or more detection platforms alone or in combination. For miRNA profiling other factors also need to be considered such as whether the sample was digested with DNase or not, and the cDNA input and synthesis method. In the case of RT-qPCR, normalization must be performed with reference genes

With the growing interest in miRNAs as biomarkers for BFID in forensic science, a variety of platforms have been employed to analyse miRNA expression in body fluids. MiRNA profiling is a multi-step process that typically includes the selection of miRNA markers, body fluid collection, nucleic acid extraction, cDNA synthesis, and quantitative PCR (qPCR). Current techniques for miRNA detection include northern blotting, microarray analysis, capillary electrophoresis, quantitative reverse transcription PCR (RT-qPCR), and next-generation sequencing (Fig. 1).

However, the candidate miRNAs studied to date rarely overlap between research groups, leading to inconsistent methods and protocols, and thus a lack of reproducibility and replicability of miRNAs from target body fluids. One potential cause for this discrepancy is the use of different combinations of methods for BFID by various research teams. This issue was highlighted by Zubakov et al., [23], who were unable to replicate the results of Hanson et al., [22] and attributed this failure to differences in the techniques used.

Given these challenges, it is increasingly evident that standardization and optimization of miRNA profiling methods are essential. A reliable biomarker must maintain stability during and after the isolation process to allow for accurate detection and quantification. However, it has been observed that miRNA recovery can vary significantly depending on the extraction technique employed [43–51].

Several methods have been developed for miRNA isolation, with commercial kits such as miRNeasy (Qiagen), PureLink (Invitrogen), and mirVana (Ambion) being widely used in forensic BFID and other biological research areas [52]. In response to the discrepancies observed across studies, research groups from various fields, including biology and medicine, have initiated comparisons of different extraction methods to assess their impact on miRNA recovery [43–47, 50, 51, 53]. Some studies have further suggested that miRNA recovery may be influenced by factors such as GC content, thermostability, and the length of RNA molecules [45, 47, 53].

This study is a proof-of-concept aimed at evaluating two aspects: first, to evaluate the efficiency of different extraction methods for simultaneously isolating (co-isolation) DNA, RNA, and miRNA from human saliva; and second, to determine which extraction protocol yields the highest miRNA levels detected with the lowest quantification cycle (Cq) when analysed with RT-qPCR.

## Materials and methods

This research employs two DNA extraction kits, and one widely used miRNA isolation kit to extract nucleic acids, including DNA, RNA, and miRNAs, from human saliva samples. Human saliva was chosen due to its frequent presence at crime scenes and the relative ease of sample collection. The central objective of this study is to develop a streamlined co-extraction method that enables both DNA profiling and BFID from a single sample source without compromising the quality or quantity of the extracted nucleic acids. Given that commercially available miRNA isolation kits are designed to be highly selective and specific for miRNAs, one such kit was compared against two genomic DNA extraction kits.

Additionally, we investigated the impact of sample input volume on nucleic acid extraction efficiency by using saliva samples of five different volumes, decreasing by a factor of two for each subsequent sample. This aspect of the study is particularly relevant because recovering high-quality and high-quantity body fluid samples from real crime scenes is often challenging.

For quantification, two detection platforms, NanoDrop and Qubit, were utilized. Since NanoDrop cannot specifically target and quantify miRNA, Qubit was used for miRNA quantification. Additionally, a two-step RT-qPCR was performed to assess miRNA expression levels in the extracted body fluid samples.

**Fig. 2:**
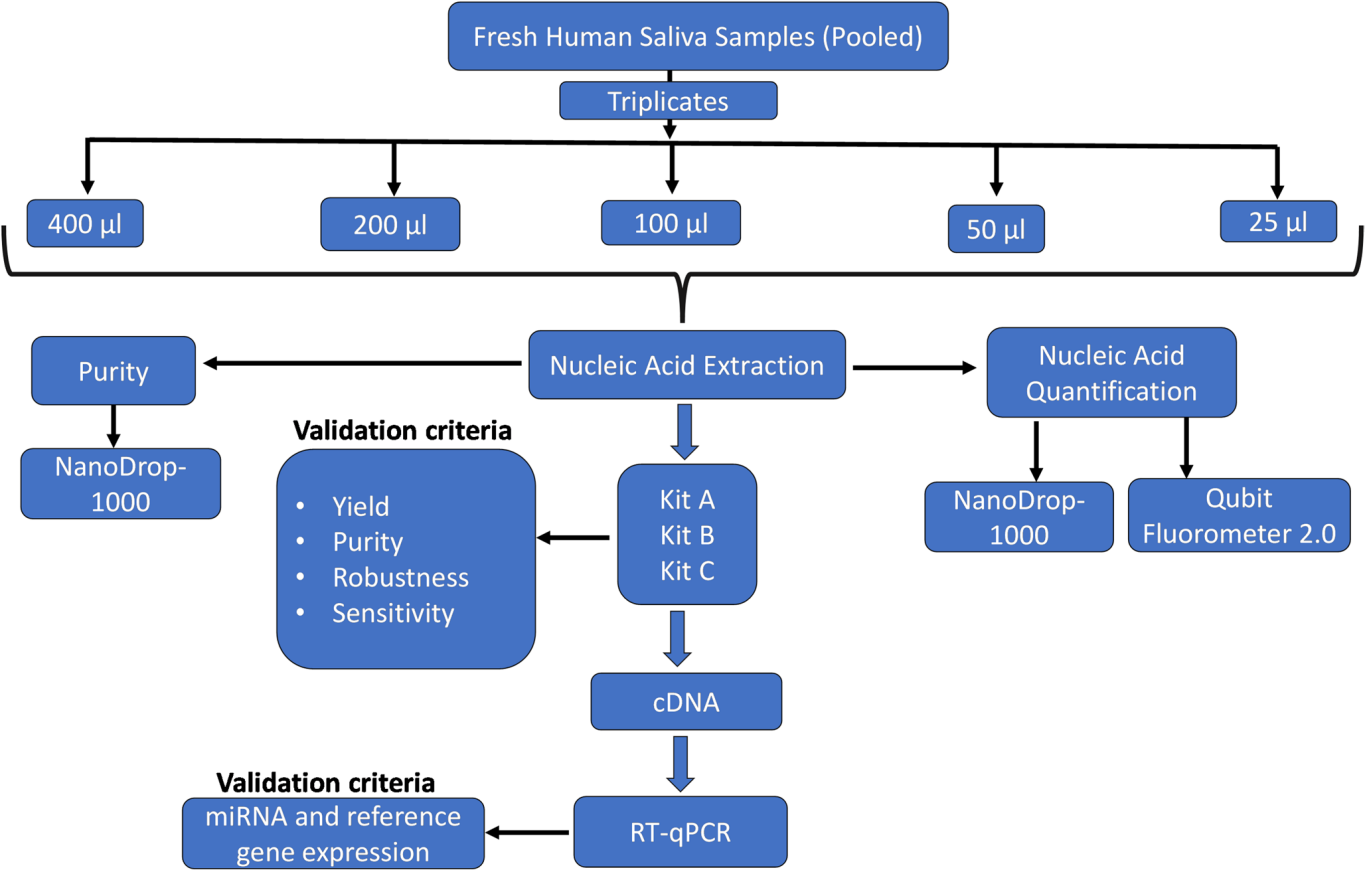
Overview of experimental design. Saliva collected from three participants (*N* = 3) were pooled. Nucleic acids were extracted using three different kits: Kit A- AccuPrep^®^ Genomic DNA Extraction Kit, Kit B -Qiamp DNA Mini Kit (Qiagen) and Kit C - miRNeasy Tissue/Cells Advanced Micro Kit (Qiagen). The quantification was performed using two different methods. Additionally, miRNA and reference gene expression levels were then compared using RT-qPCR

The overall research workflow is outlined in Fig. 2.

### Sample collection

Human saliva samples were collected from three healthy volunteers over 18 years of age with informed consent, following ethical approval. Volunteers were asked to avoid eating and drinking for at least 30 min before sample collection and saliva samples were collected by spitting into a 15 mL centrifuge tube.

### Nucleic acid extraction

To minimise bias due to inter-sample heterogeneity, 3 mL saliva from each individual sample was pooled together, and the pooled sample was then split into five different volumes: 400 µL; 200 µL; 100 µL; 50 µL and 25 µL. Three technical replicates of each volume were then extracted with three different spin-column-based extraction kits, namely, AccuPrep Genomic DNA Extraction Kit (Bioneer Corporation, Republic of Korea), QIAamp DNA Mini Kit (Qiagen, Germany) and miRNeasy Tissue/Cells Advanced Micro Kit (Qiagen, Germany). All extractions were performed according to the manufacturers’ protocols, using the provided elution buffers. A final elution volume of 50 µL was used for all kits, and no DNase digestion was performed. Following extraction and elution, samples were immediately stored at −20 °C.

### Nucleic acid quality and quantity assessment

A NanoDrop spectrophotometer (Thermo Fisher Scientific) was used for DNA and RNA quantification. Purity was measured in terms of A260/A280 and A260/A230 absorbance ratio according to Beer-Lambert’s law. A 260/280 ratio of ≥ 1.8 and 260/230 ratio in the range of 2.0- 2.2 is considered to reflect pure DNA or RNA [47]. First, the instrument was blanked with 1 µL of the same elution buffer that was used during extraction procedure for elution. Following the manufacturer’s instructions, 1 µL of sample was used to measure the concentration (ng/µL) and purity of each sample.

A Qubit 2.0 Fluorometer (Thermo Fisher Scientific) was used for quantification of miRNA content using a microRNA assay kit (Thermo Fisher Scientific, Eugene, Oregon, USA). Quantification assessment was conducted according to the manufacturer’s protocol using a sample volume of 1 µL per assay, and concentration (ng/mL) was measured by the instrument.

### MiRNA marker selection

Based on a literature study in the field of forensic BFID (Table 1), the following miRNAs and reference genes were selected. MiR-205 and miR-203 are considered saliva-specific markers while RNU6b, RNU44, and RNU48 are the most commonly used reference genes in forensic BFID research. Information regarding each miRNA and reference gene is given in Table 2.

**Table 1:** List of potential body fluid-specific MiRNA markers identified by various authors, for BFID

Blood-specific markers	Menstrual blood-specific	Saliva-specific markers	Semen-specific markers	Vaginal- specific secretion markers	Skin-specific markers	Reference genes
miR-451 [22, 34, 37] miR-16 [36, 39, 54] miR-126 [34, 55] miR-144-3p [27, 55–57] miR-144-5p [56]	miR-451 [22, 28, 58] miR-412 [22, 29, 59] miR-214 [31, 54, 58] miR-144-3p [39, 42, 55, 60] miR-144-5p [39, 42, 55, 60] miR-141-3p [26, 29, 55, 61] miR-185-5p [39, 42] miR-497-5p [ 26, 55, 61]	miR-205 [22, 34, 38, 40] miR-203 [34, 59, 61] miR-200c [34, 56] miR-658 [22, 39] miR-145-5p [41] miR-223-3p [41, 55–57]	miR-891a [23, 27, 39, 54] miR-888 [26, 30, 54, 57] miR-10b [29, 30, 62] miR-10a [ 30, 61, 63] miR-135a [39] miR-135b [23]	miR-124a[22, 27, 31, 54] miR-654-5p [31, 41]	miR-3169 [28, 39]	RNU48 [23] RNU44 [23, 40] RNU6b [31, 34, 60, 62]

**Table 2:** Details of each MiRNA and reference gene used in this study

Body Fluid	Assay Name	Sequence	Assay ID	miRBase Accession Number
Saliva	hsa-miR-205	UCCUUCAUUCCACCGGAGUCUG	000509	MI0000285
	hsa-miR-203	GUGAAAUGUUUAGGACCACUAG	000507	MI0000283
				NCBI Accession Number
Reference genes	RNU44	CCTGGATGATGATAGCAAATGCTGACTGAACATGAAGGTCTTAATTAGCTCTAACTGACT	001094	NR_002750
	RNU6b	CGCAAGGATGACACGCAAATTCGTGAAGCGTTCCATATTTTT	001093	NR_002752
	RNU48	GATGACCCCAGGTAACTCTGAGTGTGTCGCTGATGCCATCACCGCAGCGCTCTGACC	001006	NR_002745

### Reverse transcription and quantitative real time PCR (RT-qPCR)

A two-step RT-qPCR approach was used for the analysis of miRNA. This involved the synthesis of complementary DNA (cDNA) and quantification of the cDNA using real-time PCR. The optimization of the RT-qPCR protocol is explained below.

#### Complementary DNA (cDNA) synthesis

For the validation of the expression of selected miRNA markers and reference genes, the pre-designed TaqMan RT-PCR assay was used. cDNA was synthesized using the TaqMan MicroRNA Reverse Transcription Kit (Thermo Fisher Scientific, Vilnius, Lithuania) and target-specific stem-loop primers (Thermo Fisher Scientific, Pleasanton, CA, USA). The “TaqMan^®^ Small RNA Assays” protocol was used and optimized for cDNA synthesis.

A master mix (RT reaction mix) was prepared by adding 100 mM dNTPs, 50 U/µL MultiScribe Reverse Transcriptase, 10 X Reverse Transcription Buffer, 20 U/µL RNase Inhibitor and Nuclease-free water in an appropriately sized microcentrifuge tube. The 3.5 µL of RT reaction mix, and 2.5 µL extract/template were first combined in an Eppendorf tube or PCR tube (0.2 mL), then mixed thoroughly and briefly centrifuged. After that, 1.5 µL 5 X RT primer was added to each reaction tube. Reverse transcription reactions were performed in a final volume of 7.5 µL. A total of 10 ng RNA input was used for cDNA synthesis in samples extracted with the AccuPrep Genomic DNA Extraction Kit (Bioneer) and the miRNeasy Tissue/Cells Advanced Micro Kit (Qiagen), while 8.5 ng total RNA was used for the QIAamp DNA Mini Kit (Qiagen) due to lower RNA recovery. The reverse transcription was performed at 16 °C for 30 min, 42 °C for 30 min and 85 °C for 5 min with a final hold at 4 °C in a thermocycler (Techne Prime Thermal Cycler). The RT reaction product was immediately stored at −20 °C until further analysis. A negative control (i.e. no reverse transcriptase) was also included for each sample.

#### Quantitative PCR (qPCR)

The “TaqMan^®^ Small RNA Assays”, as used in the cDNA synthesis, was also used to perform real-time PCR amplification. To perform PCR amplification, a PCR Reaction Mix was prepared in an appropriately sized microcentrifuge tube. A single PCR Master mix containing 20 X TaqMan^®^ Small RNA Assay, PCR Master Mix II with no UNG (Thermo Fisher Scientific, Vilnius, Lithuania) and nuclease-free water (Ambion, USA) was mixed and then briefly centrifuged. The final reaction volume was 10 µL, comprising 0.67 µL of cDNA template and 9.34 µL of PCR Reaction Mix. All sample assay combinations were prepared in triplicate by individual sample, in an optical 96-well reaction plate (Applied Biosystems™) and sealed with clear adhesive film (Applied Biosystems™). Two negative controls (i.e. no template and no reverse transcriptase) were also incorporated for each sample in the plate to detect any false positive amplification due to cross-contamination of reagents, primers or genomic DNA. Quantitative PCR was performed using a QuantStudio 5 instrument (Applied Biosystem) with the following PCR cycling parameters: 95 °C for 10 min; followed by 40 cycles of denaturation and annealing at 95 °C for 15 s, and 60 °C for 60 s, respectively; and an extension of the template during the temperature ramp of annealing and denaturation steps. The Cq values were automatically calculated by the Design and Analysis Software 2.6.0. However, the software was optimally calibrated, and certain criteria (explained below) were kept in place to study the expression of miRNA in specific body fluids.

#### Optimization of RT-qPCR data analysis software

For the study of target miRNA and reference gene levels detected in body fluid, Presence/Absence Analysis software available in QuantStudio 5 was used. The presence/absence analysis method is used to make call i.e., presence or absence of a target nucleic acid sequence in a sample (Applied Biosystem Presence Absence Analysis Module User Guide).

The following settings were adjusted in the software: baseline setting (signal level during the initial cycles of PCR) – 3–15; and threshold value (the level of fluorescence above the baseline) – 0.2. Cq was set at < 30 for miRNA and < 36 for reference genes.

#### Statistical analysis

GraphPad Prism was used to calculate the median, range, coefficient of variation (CV%), and linear regression to evaluate nucleic acid yield, purity, robustness, and sensitivity for each extraction method. Statistical significance was assessed using the Friedman test for each extraction method and the Kruskal-Wallis test for miRNA expression levels, both applied with a significance level of 0.05, using GraphPad Prism 8.0.2. For miRNA expression analysis, raw Cq values mean, Cq values, and Cq standard deviation were calculated using QuantStudio 5 - Design and Analysis Software 2.6.0.

## Results

The nucleic acid extraction protocol was validated for:

**Performance**, evaluated in terms of quantity and quality of nucleic acid as well as miRNA expression.

- **Quantity**, assessed in terms of each nucleic acid yield, i.e., DNA, RNA, and miRNA.
- **Quality**, analysed in terms of purity.
- **miRNA level detected**, assessed as effect of kit on miRNA level detected, evaluated in terms of Cq values. The lower the Cq value, the higher is the level of miRNA detected.

**Robustness**, evaluated as the ability of the extraction method to give constant yield of nucleic acid, irrespective of sample volumes.

**Sensitivity**, assessed as how well the extraction method responds to changes in the sample volume. Specifically, how much the nucleic acid yield changes upon varying the volume of the sample.

### Evaluation of DNA isolation using three extraction methods

**Fig. 3:**
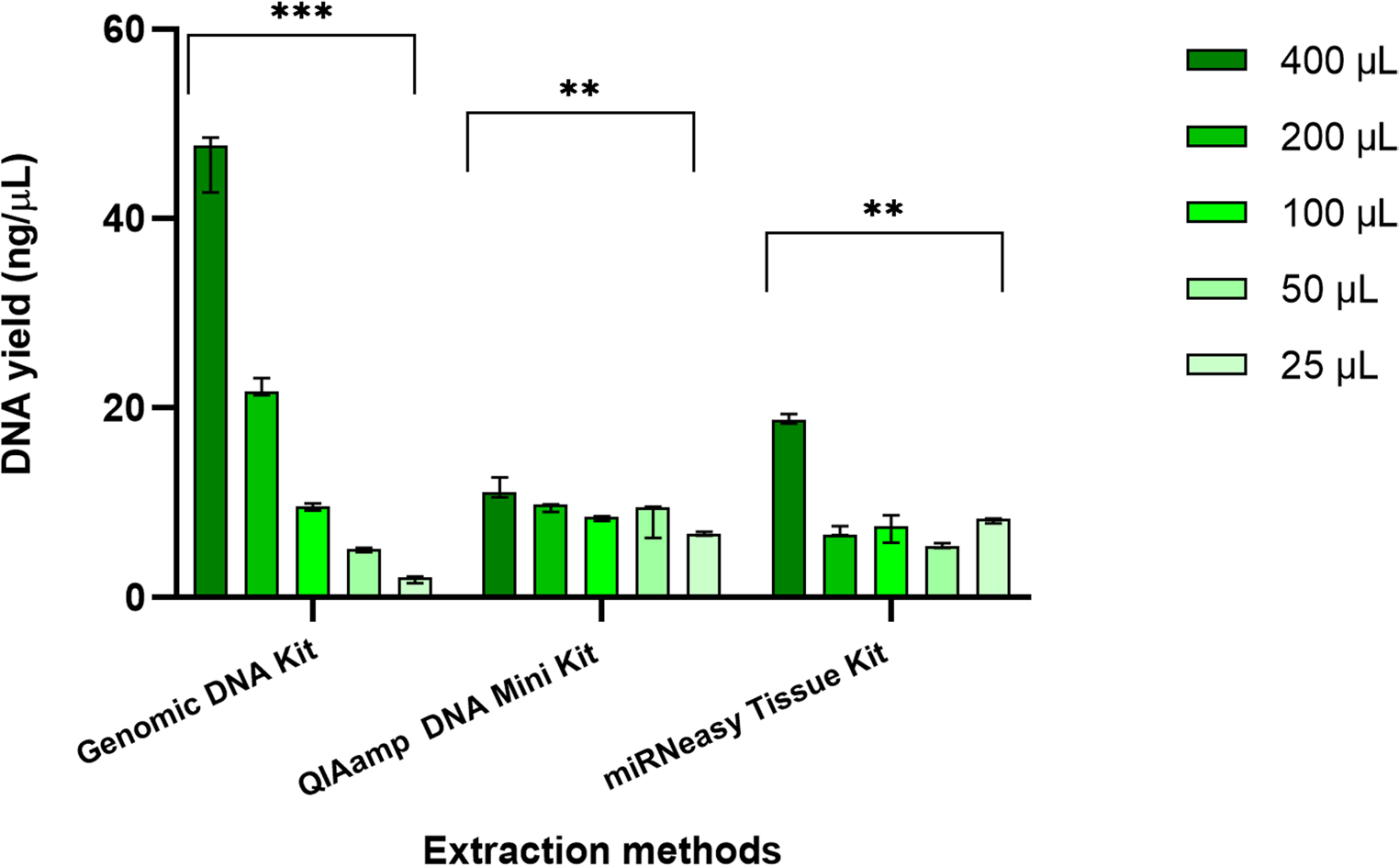
Comparison of yield of DNA from 400 µL, 200 µL, 100 µL, 50 µL and 25 µL human saliva samples (in triplicates) extracted using three different extraction kits i.e., AccuPrep^®^ Genomic DNA Extraction Kit, Qiagen QIAamp DNA Mini Kit, and Qiagen miRNeasy Tissue/Cells Advanced Micro Kit. Quantification of DNA was performed using a NanoDrop-1000. Error bars represent the range of values from technical replicates. Statistical significance is indicated as follows: ***P < 0.001, **P < 0.01

The bar graph in Fig. 3 illustrates the comparative DNA yield obtained from three different extraction kits (Bioneer AccuPrep^®^ Genomic DNA Extraction Kit, Qiagen QIAamp DNA Mini Kit, and Qiagen miRNeasy Tissue/Cells Advanced Micro Kit) across five saliva sample volumes (400 µL, 200 µL, 100 µL, 50 µL, and 25 µL).

**Fig. 4:**
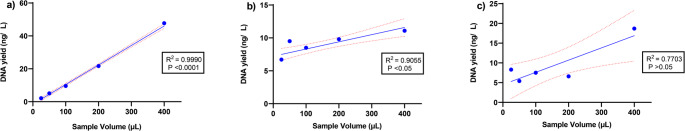
Sensitivity of nucleic acid extraction methods to changes in sample volume. The x-axis represents the sample volume (25 µL, 50 µL, 100 µL, 200 µL, 400 µL) and the y-axis represents the DNA yield. Each data point represents the median DNA yield from three replicate experiments for the given sample volume. The R² value indicates the strength of the relationship between input volume and DNA yield, reflecting how well each extraction method responds to changes in sample volume **(A)** A stron, statistically significant linear relationship was observed for the Bioneer AccuPrep^®^ Genomic DNA Extraction Kit, with 99.90% of the variability in DNA yield explained by input volume. This indicates consistent DNA extraction across varying input volumes **(B)** A statistically significant linear relationship was observed for the QIAamp DNA Mini Kit with 90.55% of the variability in DNA yield explained by input volume, demonstrating consistent performance across varying volumes **(C)** A non-significant relationship was observed for the miRNeasy Tissue/Cells Advanced Micro Kit, with only 77.03% of the variability in DNA yield explained by input volume. This suggests inconsistent DNA extraction across varying sample volumes

The Genomic kit consistently produced the highest DNA yields, particularly from larger sample volumes: 47.70 ng/µL at 400 µL with range 5.80 ng/µL, but its effectiveness decreased with smaller volume i.e., 25 µL (2.10 ng/µL with range 0.70 ng/µL). Despite high yields, the Genomic kit showed variability in purity; the A260 nm/A280 nm ratios for the Genomic kit ranged from 2.76 to 1.49, with no significant differences in 260/280 (*P* = 0.29) between all sample volumes (Table 3). The extraction method was found be robust with CV%: CV less than 5% for 100 µL and 200 µL, and CV less than 10% for volumes under 100 µL. However, at the smallest volume of 25 µL, the CV was significantly larger (19.58%), reflecting reduced consistency in DNA yield and decreased robustness (Table 3). The Genomic kit was found to be highly sensitive, with an R^2^ value of 0.9990; the regression model was highly significant, with a P-value of < 0.0001, indicating that changes in sample volume have a significant impact on DNA yield (Fig. 4A). These results demonstrate that the Genomic kit exhibits high sensitivity for DNA extraction, with a consistent relationship between sample volume and DNA yield.

**Table 3:** Robustness and purity of DNA isolated using Bioneer AccuPrep® Genomic DNA Extraction Kit, Qiagen QIAamp DNA Mini Kit, and Qiagen miRNeasy Tissue/Cells Advanced Micro Kit from human saliva samples of 400 μL, 200 μL, 100 μL, 50 μL, and 25 μL

		Robustness of DNA Yield			DNA Purity (A260nm/A280nm ratio)				DNA Purity (A260nm/A230nm ratio)			
Extraction Method	Sample Volume (µL)	CV%	Acceptance	P-value	Mean	SD	P-value		Mean	SD	P-value	
Genomic DNA Kit	400	6.79%	CV < 10%	< 0.0001	1.86	0.03	0.29		1.80	0.15	0.10	
	200	4.29%	CV < 5%		1.68	0.12			1.67	0.46		
	100	3.67%	CV < 5%		2.08	0.27			1.22	0.42		
	50	4.14%	CV < 5%		2.76	0.74			1.61	0.73		
	25	19.58%	CV < 20%		1.49	0.90			0.55	0.16		
QIAamp DNA Kit	400	9.57%	CV < 10%	<0.01	1.49	0.02	0.48		2.18	0.88	0.25	
	200	4.85%	CV < 5%		1.86	0.38			1.76	0.87		
	100	3.15%	CV < 5%		1.86	0.69			1.00	0.76		
	50	22.17%	CV < 30%		1.72	0.58			2.21	1.30		
	25	2.99%	CV < 5%		1.36	0.14			0.90	0.39		
miRNeasy Tissue Kit	400	2.68%	CV < 5%	< 0.01	2.13	0.37	0.72		0.14	0.09	0.22	
	200	7.53%	CV < 10%		2.15	0.25			0.05	0.04		
	100	19.87%	CV < 20%		2.10	0.42			0.04	0.02		
	50	4.63%	CV < 5%		1.89	0.31			0.04	0.02		
	25	3.54%	CV < 5%		1.85	0.41			0.080	0.04		

CV% Coefficient of Variation, *SD *Standard Deviation, *µL * Microliters, *Mean *Average, *P*-value Probability value

The Qiagen QIAamp DNA Mini Kit yielded significantly lower DNA concentrations compared to the Genomic DNA Extraction Kit; at 400 µL, the median yield was 11.10 ng/µL (2.10 ng/µL variability), and at 25 µL, it was 6.70 ng/µL (range 0.40 ng/µL) with low yield. The A260 nm/A280 nm ratios for the QIAamp kit ranged from 1.36 to 1.86, with values close to 1.8 suggesting relatively pure DNA (Table 3). When robustness of DNA extraction using the QIAamp kit was evaluated, results indicated that kit performed most consistently at 25 µL (CV = 2.99%) and 100 µL (CV = 3.15%), demonstrating high robustness at these volumes, while performance at 400 µL (CV = 9.57%) showed increased variability (Table 3). The least robust performance was observed at 50 µL (CV = 22.17%). An R² value of 0.9055 was observed with significance (*P*-value < 0.05), confirming that extraction method is sensitive to sample volume, which thus has a direct impact on DNA yield (Fig. 4B).

The Qiagen miRNeasy Tissue/Cells Advanced Micro kit exhibited a moderate range in DNA yield, with the highest yield being 18.70 ng/µL at 400 µL, with range 1.00 ng/µL and the lowest yield being 5.40 ng/µL (0.50 ng/µL) at 50 µL. The miRNeasy kit performed better than the QIAamp kit in terms of yield at higher sample volumes with low variability. The range values were generally low, compared to the Genomic kit and QIAamp kit. The A260 nm/A280 nm ratios for the miRNeasy kit ranged from 1.85 to 2.15, generally indicating higher purity compared to the Genomic and QIAamp kits, suggesting that miRNeasy kit might be more effective in removing protein contaminants (Table 3). Statistical analysis revealed no significant differences in A260 nm/A280 nm ratios (*P* = 0.72) between different sample volumes. The highest robustness was observed at 400 µL, 25 µL, and 50 µL where CV% was < 5%, but at 100 µL, CV% was 19.87% (Table 3). The miRNeasy kit demonstrated a moderate sensitivity to changes in sample volume, with an R² value of 0.7703, and a slightly significant effect on DNA yield (*P* = 0.0504) (Fig. 4C).

These findings suggest that the Genomic extraction kit is the most efficient in terms of yield and sensitivity, making it suitable for applications requiring high DNA concentrations, though variability in purity was observed. The QIAamp kit offers consistent and reliable results but with lower yields. The miRNeasy kit provides moderate yields with generally high purity from protein. Statistical analysis indicated significant differences in yield and purity among the kits mostly with *P* < 0.05.

### Evaluation of RNA isolation using three extraction methods

**Fig. 5:**
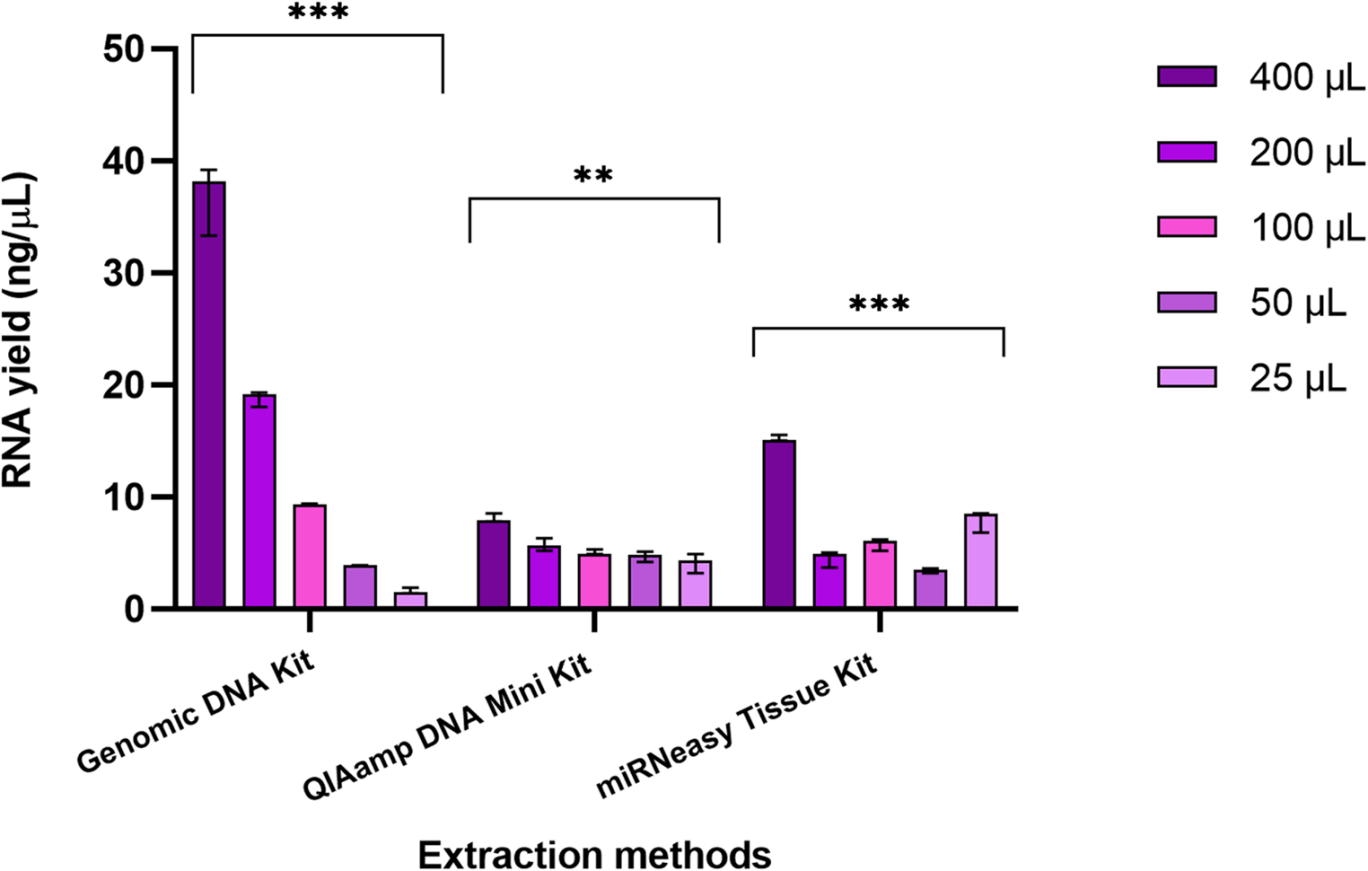
Comparison of yield of RNA from 400 µL, 200 µL, 100 µL, 50 µL and 25 µL human saliva samples (in triplicates) extracted using three different extraction kits, i.e., AccuPrep^®^ Genomic DNA Extraction Kit, Qiagen QIAamp DNA Mini Kit, and Qiagen miRNeasy Tissue/Cells Advanced Micro Kit. Quantification of RNA yield was done using a NanoDrop-1000. Error bars represent the range of technical replicates. Statistical significance is indicated as follows: ***P < 0.0001, **P < 0.01

The comparative analysis of RNA yield and purity from saliva samples using three extraction kits (Fig. 5) across various sample volumes revealed notable differences in performance.

The Genomic kit consistently provided the highest RNA yields across all sample volumes, with a peak median yield of 38.20ng/µL at 400 µL (range 5.90 ng/µL) to 1.50 ng/µL at 25 µL (range 0.50 ng/µL). However, the purity of RNA yielded varied, with A260 nm/A280 nm ratios ranging from 1.70 to 2.55, indicating some protein contamination. Statistical analysis revealed no significant differences in A260 nm/A280 nm ratio (*P* = 0.12). Despite this, the Genomic kit produced generally acceptable RNA purity, especially at larger volumes (Table 4).

**Table 4:** Robustness and purity of RNA isolated using the Bioneer AccuPrep^®^ genomic DNA extraction kit, Qiagen QIAamp DNA mini kit, and Qiagen MiRNeasy Tissue/Cells advanced micro kit from human saliva samples of 400 µL, 200 µL, 100 µL, 50 µL, and 25 µL

		Robustness of RNA Yield			RNA Purity (A260nm/A280nm ratio)			RNA Purity(A260nm/A230nm ratio)		
Extraction Method	Sample Volume (µL)	CV%	Acceptance	P-value	Mean	SD	P-value	Mean	SD	P-value
Genomic DNA Kit	400	8.56%	CV < 10%	< 0.0001	1.86	0.01	0.12	2.05	0.03	0.03
	200	3.84%	CV < 5%		1.90	0.03		1.86	0.24	
	100	0.62%	CV < 5%		1.70	0.39		1.09	0.16	
	50	1.49%	CV < 5%		2.55	0.22		1.10	0.25	
	25	16.54%	CV < 20%		1.72	0.83		0.68	0.12	
QIAamp DNA Kit	400	4.69%	CV < 5%	<0.001	2.27	0.15	0.72	1.66	0.45	0.72
	200	9.61%	CV < 10%		1.79	0.31		1.78	0.59	
	100	4.59%	CV < 5%		2.44	0.30		1.09	0.68	
	50	9.75%	CV < 10%		2.24	0.41		1.99	0.68	
	25	20.86%	CV < 30%		1.90	0.37		1.02	0.56	
miRNeasy Tissue Kit	400	1.74%	CV < 5%	< 0.0001	2.05	0.15	0.68	0.14	0.04	0.05
	200	15.96%	CV < 20%		2.00	0.10		0.05	0.02	
	100	9.44%	CV < 10%		2.18	0.29		0.04	0.02	
	50	6.06%	CV < 10%		2.41	0.07		0.03	0.01	
	25	12.37%	CV < 20%		2.09	0.37		0.10	0.02	

*CV% *Coefficient of Variation*, SD *Standard Deviation*, µL *Microliters*, Mean Average, P-value *Probability value

**Fig. 6:**
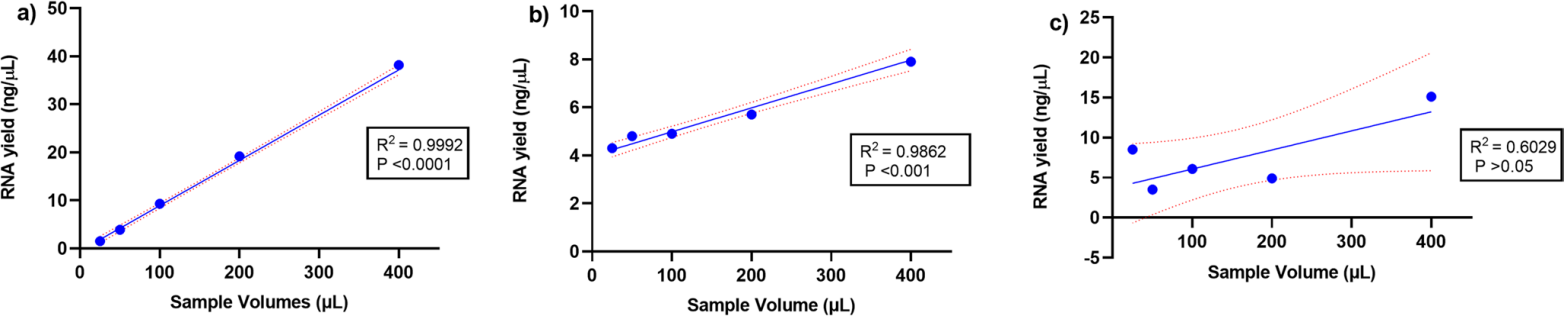
Sensitivity of nucleic acid extraction methods to changes in sample volume. The x-axis represents the sample volume (25 µL, 50 µL, 100 µL, 200 µL, 400 µL) and the y-axis represents the RNA yield. Each data point represents the median RNA yield from three replicate experiments for the given sample volume. The R² value indicates the strength of the relationship between input volume and RNA yield, reflecting how well each extraction method responds to changes in sample volume **(A)** A strong, statistically significant linear relationship was observed for the AccuPrep^®^ Genomic DNA Extraction Kit, with 99.92% of the variability in RNA yield explained by input volume. This indicates consistent RNA extraction across varying input volumes **(B)** A statistically significant linear relationship was observed for the QIAamp DNA Mini Kit, with 98.62% of the variability in RNA yield explained by input volume, demonstrating consistent performance across varying volumes **(C)** A non-significant relationship was observed for the miRNeasy Tissue/Cells Advanced Micro Kit, with only 60.29% of the variability in RNA yield explained by input volume. This suggests inconsistent RNA extraction across varying sample volumes

The robustness of RNA extraction by the Genomic kit was as follows: CV% values were below 5% for 200 µL, 100 µL, and 50 µL input volumes, indicating high consistency and robust performance at these levels. However, at the smallest input volume (25 µL) CV% was 16.54%, indicating reduced robustness and increased variability (Table 4). The sensitivity of the Genomic kit for RNA yield and input volume was found to be strong when measured, with an R² value of 0.9992. The relationship was statistically significant (*P* < 0.0001), highlighting the kit’s high sensitivity and consistent performance across the tested range of sample volumes (25–400 µL) (Fig. 6A), where the RNA yield is directly proportional to the amount of sample used.

The highest median RNA yield of the QIAamp kit was obtained at the largest sample volume of 400 µL (7.90 ng/µL) with range 0.70 ng/µL, while the smallest volume of 25 µL produced the lowest median yield (4.30 ng/µL) with the largest range (1.700 ng/µL) thus showing lower consistency in RNA yield at smaller volumes. The QIAamp kit exhibited A260 nm/A280 nm ratios ranging from 1.79 to 2.44, with values closer to 2.0 suggesting relatively pure RNA (Table 4); statistical analysis revealed no significant differences in A260 nm/A280 nm ratios (*P* = 0.72). Robustness was indicated by the low CV values (< 5%) of 4.69% and 4.59% at 400 µL and 100 µL, respectively. However, robustness was decreased at 25 µL, where the CV value was relatively high (> 20%) i.e., 20.86% (Table 4). The sensitivity analysis revealed a strong positive linear relationship between sample volume and RNA yield with an R² value of 0.9862; the relationship was statistically significant (*P* < 0.001) (Fig. 6B).

The miRNeasy Kit provided moderate RNA yields. A median yield of 15.10 ng/µL with a range of 0.50 ng/µL was observed at 400 µL. Yield was lower at 200 µL (4.90 ng/µL) and at 50 µL (3.50 ng/µL), and then higher at 25 µL (8.50 ng/µL) with range 1.70 ng/µL. The miRNeasy kit showed A260 nm/A280 nm ratios ranging from 2.00 to 2.41, generally indicating higher purity compared with the Genomic DNA extraction and QIAamp kits. Statistical analysis revealed no significant differences in A260 nm/A280 nm ratios (*P* = 0.68) (Table 4). High robustness and consistency in RNA yield was observed at the largest sample volume (400 µL), with a low CV (< 5%). However, at smaller sample volumes (200 µL, 100 µL, 50 µL, and 25 µL), the CV was between 10%−20% indicating a drop in robustness (Table 4). The sensitivity of the miRNeasy kit was found to be poor for small sample volumes, which is indicated by the non-significant slope (P-value = 0.12), and the moderate R² value (0.6029), demonstrating that a large proportion of the variability in RNA yield cannot be explained by sample volume alone, i.e. the kit is less sensitive to sample volume input (Fig. 6C).

 Significant differences in RNA yield (*P* < 0.05) were observed among the kits and sample volumes, with the Genomic DNA extraction kit providing the highest yield across all volumes tested. The QIAamp and miRNeasy kits showed high variability and less consistency in yield and purity compared to the Genomic kit.

### Evaluation of miRNA isolation using three extraction methods

**Fig. 7:**
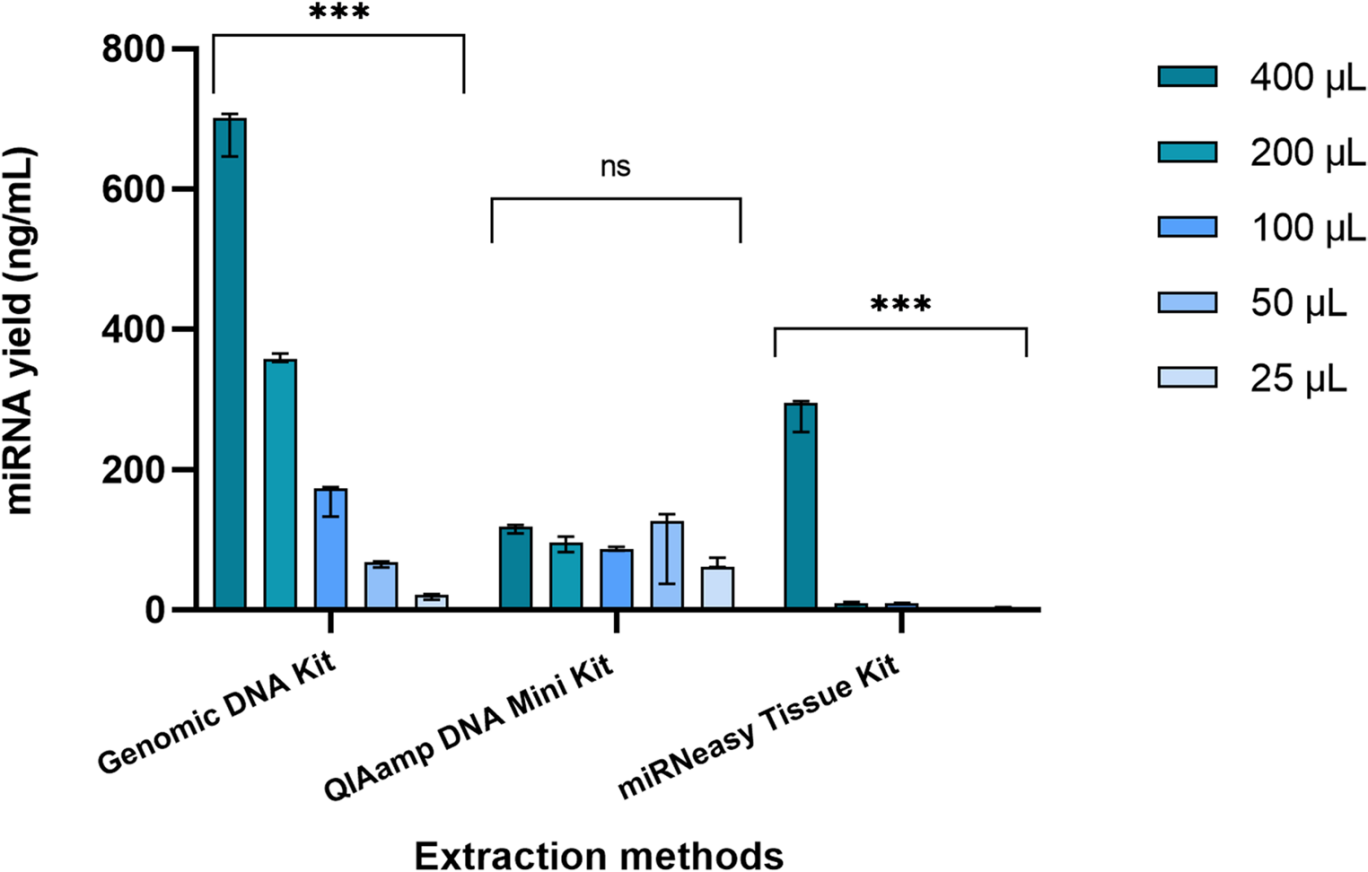
Comparison of yield of miRNA from 400 µL, 200 µL, 100 µL, 50 µL and 25 µL human saliva samples (in triplicates) extracted using three different extraction kits i.e., AccuPrep® Genomic DNA Extraction Kit, Qiagen QIAamp DNA Mini Kit, and Qiagen miRNeasy Tissue/Cells Advanced Micro Kit. Quantification of miRNA yield was done using a Qubit Fluorometer. Error bars represent the range of technical replicates. Statistical analysis showed a highly significant difference for the AccuPrep and QIAamp extraction kits (***P < 0.0001), while results for the miRNeasy Tissue/Cells kit were not significant (ns)

 The miRNA yield from the three extraction kits across five saliva sample volumes revealed distinct performance patterns (Fig. 7). 

**Fig. 8:**
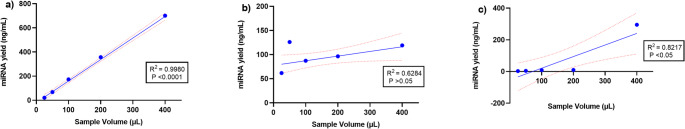
Sensitivity of nucleic acid extraction methods to changes in sample volume. The x-axis represents the sample volume (25 µL, 50 µL, 100 µL, 200 µL, 400 µL) and the y-axis represents the miRNA yield. Each data point represents the median miRNA yield from three replicate experiments for the given sample volume. The R² value indicates the strength of the relationship between input volume and miRNA yield, reflecting how well each extraction method responds to changes in sample volume **(A)** A strong, statistically significant linear relationship was observed for the AccuPrep^®^ Genomic DNA Extraction Kit, with 99.80% of the variability in miRNA yield explained by input volume. This indicates consistent miRNA extraction across varying input volumes **(B)** A non-significant relationship was observed for the QIAamp DNA Mini Kit, with only 62.84% of the variability in miRNA yield explained by input volume, demonstrating inconsistent performance across varying volumes **(C)** A significant linear relationship was observed for the miRNeasy Tissue/Cells Advanced Micro Kit, with 82.17% of the variability in RNA yield explained by input volume. However, the graph does not strongly emphasize sensitivity to smaller changes in sample input volume, particularly at the lower end (e.g., 100 µL and below)

 The Genomic DNA extraction kit consistently delivered the highest miRNA yields, peaking at around 701.0 ng/mL for the 400 µL sample volume (range 61.00 ng/mL). Lower yields were observed across smaller sample volumes, with the lowest yield recorded at 25 µL (21.10 ng/mL) with range 8.30 ng/mL. The robustness of kit was highest at 200 µL (CV = 1.70%), followed by 400 µL (CV = 4.91%), showing high reproducibility at these volumes. The lowest robustness was observed at 25 µL (CV = 23.45%), reflecting significant variability at this small volume (Table 5). In sensitivity analysis of the Genomic kit, an R² value of 0.9980 indicated the regression model was highly significant, with P-value < 0.0001, showing consistent increases in miRNA yield as the sample volume increased (Fig. 8A).

When miRNA yield from the QIAamp kit was evaluated, the highest median yields were observed at 50 µL (126.0 ng/mL) with range of 99.20 ng/mL, followed by 400 µL (119.0 ng/mL), with range of 12.00 ng/mL. The lowest yield was recorded at 25 µL (61.70 ng/mL; range = 13.20 ng/mL). The highest robustness was observed at 100 µL (CV = 2.99%), followed by 400 µL (CV = 5.53%), However, the lowest robustness was observed at 50 µL (CV = 54.84%) (Table 5). The sensitivity of the QIAamp kit was found to not be statistically significant, as indicated by a P-value of 0.11. The analysis revealed a moderate linear relationship, with an R² value of 0.6284 (Fig. 8B).

The miRNA yield from the miRNeasy kit was surprisingly very low for most of the sample volumes except for the highest sample volume. The highest median yield, at 400 µL, was 295.0 ng/mL (range = 44 ng/mL), but a substantially lower yield was found at 200 µL (9.97 ng/mL; range = 1.06 ng/mL), with the lowest yield observed at 25 µL (2.740 ng/mL; range = 1.06 ng/mL). The lowest CV% was observed at 50 µL (2.84%), indicating high robustness and excellent consistency at this intermediate volume. Moderate robustness was observed at 400 µL (8.82%) and 100 µL (9.85%), while reduced robustness was recorded at 200 µL (22.22%) and 25 µL (19.73%), reflecting low robustness of miRNA yield at these volumes (Table 5). The linear regression analysis for the miRNeasy kit shows a significant relationship (*P* = 0.03) between sample input volume and miRNA yield, with an R² of 0.8217. However, the graph does not strongly emphasize sensitivity to smaller changes in sample input volume, particularly at the lower end (100 µL and below) (Fig. 8 C). At a higher volume (400 µL), the miRNA yield is drastically higher showing greater fluctuation compared to other sample volumes therefore, making it difficult to determine how consistently the miRNA yield responds to changes in volume across the entire range of inputs.

**Table 5:** Robustness of MiRNA isolated using the Bioneer AccuPrep^®^ genomic DNA extraction kit, Qiagen QIAamp DNA mini kit, and Qiagen MiRNeasy Tissue/Cells advanced micro kit from human saliva samples of 400 µL, 200 µL, 100 µL, 50 µL, and 25 µL

		Robustness of miRNA Yield		
Extraction Method	Sample Volume (µL)	CV%	Acceptance	P-value
Genomic DNA Kit	400	4.91%	CV < 5%	< 0.0001
	200	1.71%	CV < 5%	
	100	14.78%	CV < 20%	
	50	7.53%	CV < 10%	
	25	23.45%	CV < 30%	
QIAamp DNA Kit	400	5.53%	CV < 10%	0.21
	200	11.68%	CV < 20%	
	100	2.99%	CV < 5%	
	50	54.84%	CV > 30%	
	25	11.26%	CV < 20%	
miRNeasy Tissue Kit	400	8.82%	CV < 10%	< 0.001
	200	22.22%	CV < 30%	
	100	9.85%	CV < 10%	
	50	2.84%	CV < 5%	
	25	19.73%	CV < 20%	

*CV% *Coefficient of Variation*, µL *Microliters*, Mean *Average *P-value *Probability value

Significant differences in miRNA yield were observed among the kits and sample volumes (*P* < 0.05), with the Genomic DNA extraction kit providing the highest yield across all volumes tested except with the smallest sample volume. On the other hand, the QIAamp kit gave a consistent yield with all sample volumes tested; the results were statistically non-significant in terms of correlation with sample volume. The miRNeasy showed poor yield of miRNA with less variability compared to the Genomic and QIAamp kits.

### Effect on levels of microRNA and reference genes detected in human saliva samples extracted using different kits

**Fig. 9:**
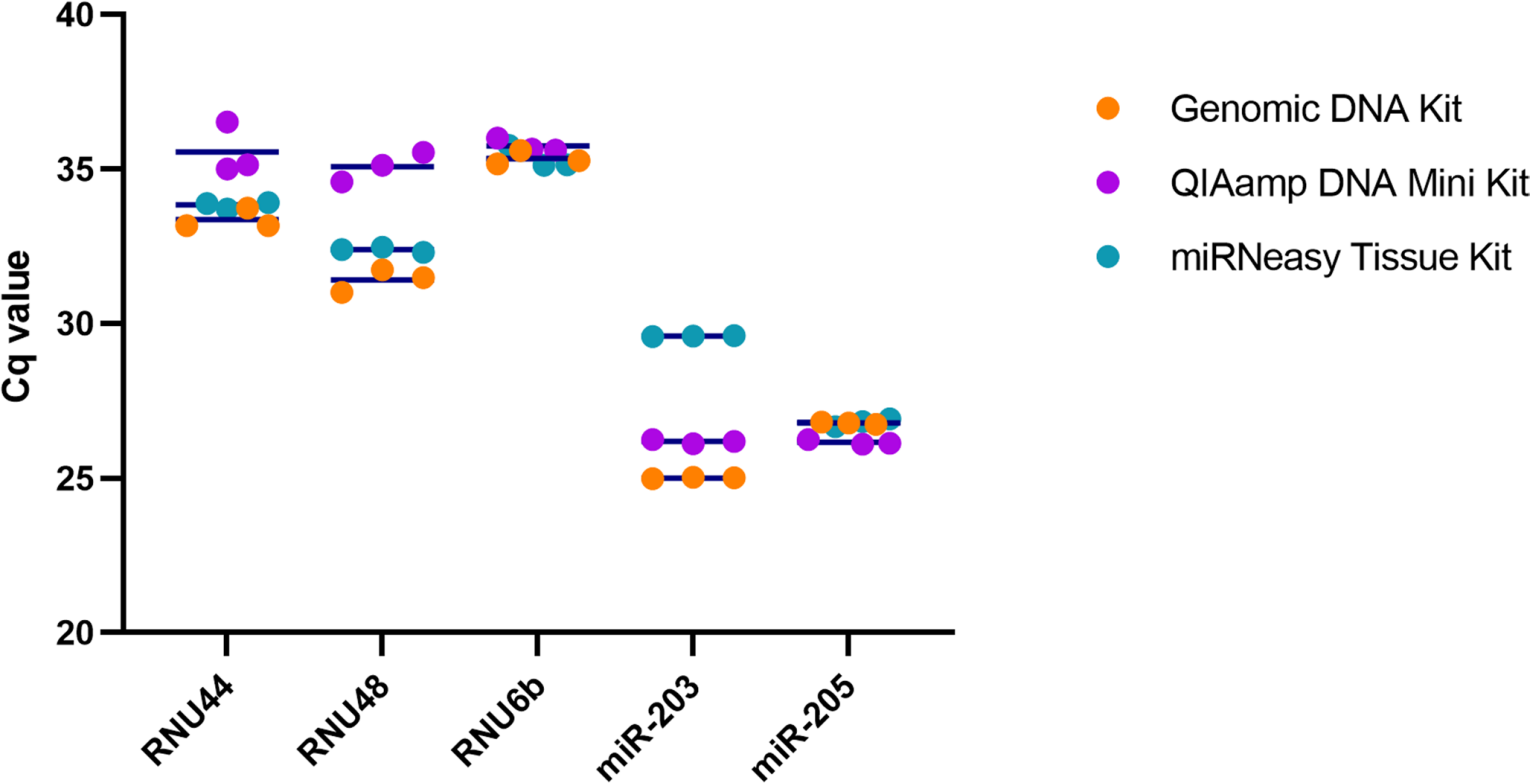
Comparison of expression levels of reference genes and miRNAs detected in human saliva sample using RT-qPCR. Saliva samples were extracted using the Genomic DNA Extraction Kit, QIAamp DNA Mini Kit, and miRNeasy Tissue/Cells Advanced Micro Kit. Individual data points represent the Cq values of technical replicates, with horizontal bars indicating the mean. Statistical differences were determined at (*P* < 0.01)

MiRNA and reference gene expression isolated from human saliva using three extraction kits without DNase digestion of the extracted samples and quantified using RT-qPCR revealed significant differences in performance between the kits. The Genomic kit consistently yielded the lowest quantification cycle (Cq) (Supplementary Table 4) values, where Cq = 25 and Cq = 26 for miRNAs (miR-203 and miR-205) respectively and for reference genes RNU44, RNU48, and RNU6b, Cq = 33.35, 31.40 and 35.33 respectively indicating the highest expression levels. This demonstrates the kit’s superior efficiency in isolating high-yield miRNAs and reference genes (Fig. 9).

In contrast, the QIAamp kit showed moderate performance with Cq values higher than the Genomic kit but lower than the miRNeasy kit. Although the levels of miR-203 and miR-205 detected were relatively consistent, they were not as high as those observed with the Genomic kit. Levels of reference genes detected followed a similar trend (Cq = 35.55, 35.07, 35.74 for RNU44, RNU48 and RNU6b, respectively). While the QIAamp kit performed adequately, its efficiency was lower than that of the Genomic kit.

The miRNeasy kit provided comparable Cq values to the Genomic kit for RNU44 and RNU48 but showed a higher Cq value for miR-203 (Cq = 29.58), indicating a lower abundance of this miRNA. However, it yielded a similar Cq value of 26.80 for miR-205, consistent with the other two kits. These results suggest that the miRNeasy kit was the least efficient in extracting high-yield miRNAs among those tested in saliva samples.

The consistency in Cq values (Cq = 35 and 26 respectively) of RNU6b and miR-205 across all kits suggests that some targets may be less sensitive to the differences in extraction methods than others. However, the variability in miR-203 (Cq ranging from 25 to 29), RNU44 and RNU48 (Cq ranging from 33 to 35 and 31 to 35, respectively) levels detected highlights the importance of selecting an appropriate extraction kit based on the specific miRNA targets of interest.

The data clearly indicate that the choice of extraction kit significantly (*P* < 0.01) impacts the levels of miRNA and reference genes detected in saliva samples. Surprisingly, the Genomic DNA extraction kit outperforms the other kits, providing the lowest Cq value consistently across all the genes studied, while the QIAamp and, especially, miRNeasy kits may be less sensitive for miRNA-based assays.

## Discussion

In this study, we observed significant impacts of nucleic acid isolation methods on the quantity, and quality of miRNA detected. The sensitivity of the extraction kits was also found to be significantly influenced by the quantity of the sample input. Similar findings have been reported by other researchers in their studies validating extraction methods [44, 47, 53], indicating that different nucleic acid isolation methods can yield different results. Therefore, optimizing the extraction protocol for result reproducibility is necessary before proceeding with downstream processing.

Based on our experiments, the Genomic kit emerged as the top-performing kit for DNA, RNA and miRNA isolation due to its high yield, sensitivity and moderate purity level. Additionally, the Genomic kit provided the best miRNA and reference gene results in terms of levels detected, characterized by the lowest point of quantification (Cq) indicating superior sensitivity and RNA integrity. Despite its overall strength, the Genomic kit exhibited a notable limitation in its performance with smaller sample volumes, where yields were significantly lower. This suggests that while the Genomic kit is well-suited for applications involving larger sample volumes, its sensitivity may be inadequate for scenarios where only minimal sample material is available, such as forensic investigations where sample quantity is often limited.

The QIAamp kit performance was deemed intermediate, exhibiting higher yields of miRNA from small sample volumes. It also produced the second-best miRNA expression results with low Cq values, but gave consistently higher Cq values for reference genes, indicating that the QIAamp kit may not be as efficient in maintaining RNA integrity, which could limit its utility in applications requiring precise quantification.

The miRNeasy kit showed promise as the second-best option for DNA and RNA isolation, yielding the highest quality of DNA and RNA. However, it failed to yield quantifiable amounts of miRNAs from small sample volumes and exhibited the highest Cq value for miR-203. This suggests that while the miRNeasy kit may be preferable for DNA and RNA applications where purity is paramount, it may not be suitable for miRNA-based studies, particularly in contexts where sample material is limited or where low-abundance miRNAs need to be detected.

It was also observed that miRNA, miR-203 and reference gene (RNU44 and RNU48) levels detected vary depending upon the type of extraction method used, whereas miR-205 and RNU6b levels detected were less sensitive to the differences in extraction methods.

The primary challenge in forensic BFID is obtaining sufficient samples, as quantities recovered from crime scenes are often limited. To address this, various co-extraction and co-analysis methods have been developed to minimize sample consumption, by some research groups [25, 38, 58].

In a study by van der Meer et al. (2013) [38], five extraction methods were investigated for co-isolating DNA and miRNA, with the QIAamp DNA Mini kit (Qiagen, UK) yielding high amounts of both. However, while this kit performed robustly in our study, it did not match the top-performing results observed by van der Meer’s group, likely due to differences in the type of kits compared, van der Meer et al. (2013) tested five, whereas our study focused on three, with only one kit in common.

Similarly, R. and Glynn (2018) [64] compared the efficiency of three extraction kits for miRNA isolation from saliva, blood, menstrual blood, vaginal material and semen. Their study found that the miRNeasy mini kit consistently yielded the highest amounts of miRNA. However, in our study, the miRNeasy tissue kit showed poorer yield with less variability compared to both the genomic and QIAamp kits, which may be due to differences in the kits compared in both studies. While both the extraction kits used in both the studies for miRNA extraction are made by Qiagen, direct comparisons are not feasible due to the different kits involved in each study. These variation underscores the complexity of selecting an optimal extraction protocol and suggests that the performance of extraction kits can vary significantly depending on the specific methods and samples used.

In our study we found that the DNA extraction kits gave the highest yield of miRNA, with lowest Cq value, compared to a specialist kit for the extraction of miRNA i.e., miRNeasy which is quite surprising. The lower yield of miRNA raises concern as most miRNAs might be lost during the extraction process, which further might affect downstream applications.

The wide range of commercial kits available in the market complicates the selection process for researchers. Preferences for extraction methods vary based on factors such as time, sample type, availability, cost, and ease of use. Additionally, miRNA profiling involves multiple stages and the use of different isolation methods and techniques, as well as different reference genes, may lead to variations in findings.

While numerous articles demonstrate the potential effectiveness of miRNAs for BFID, there is often a lack of replication of candidate miRNAs across different research groups studying the same body fluid. This inconsistency leads to low reproducibility of results. Given the use of varied isolation methods, techniques, and reference genes, it is therefore important to optimize and standardise the BFID method using miRNA markers in forensics. This optimization process can begin by standardizing the extraction and isolation of nucleic acids.

To advance the application of miRNAs in forensic BFID, there is an urgent need to optimize and standardise nucleic acid extraction protocols. Standardisation will improve reproducibility and enhance the reliability of miRNA markers in forensic investigations. The findings from this study represent a crucial step in understanding the impact of different extraction kits on nucleic acid yield and quality. However, further research involving a wider range of body fluids, larger sample sizes, diverse extraction techniques, and expanded panels of miRNAs and reference genes is necessary to develop a standardized protocol that can be reliably implemented in forensic BFID.

In our study DNA and RNA quantification were performed using NanoDrop spectrophotometry, which provides rapid and consistent measurements but may overestimate RNA concentrations in the presence of co-extracted DNA due to its non-specific detection at 260 nm [65]. While this limitation may affect the accuracy of absolute RNA quantification, NanoDrop was used consistently across all DNA and RNA samples, allowing for consistent relative comparisons of nucleic acid yield among extraction kits and sample input volumes. Future studies may benefit from incorporating RNA-specific fluorometric assays, such as Qubit, to enhance quantification accuracy.

For miRNA quantification, the Qubit fluorometric platform was used due to its higher specificity and sensitivity for small RNA species. This approach ensured more accurate assessment of miRNA yield, while downstream expression analysis was performed using two-step RT-qPCR. In conclusion, this study was conducted as a proof-of-concept to assess the efficiency of different extraction methods for the co-isolation of nucleic acids from human saliva and to evaluate the impact of three extraction kits on miRNA expression levels for forensic body fluid identification. The data presented represent a method validation. While this study offers valuable insights into the performance of various extraction kits, it also highlights the need for further comprehensive research involving additional body fluids, a broader range of extraction techniques, and improved quantification methods. 

It is essential to develop a standardised and reproducible method for miRNA profiling in forensic science. Optimizing extraction protocols is a critical step toward the broader application of miRNAs in forensic investigations, ensuring that results are reliable and reproducible across different laboratories and case scenarios.

## Supplementary Information

Below is the link to the electronic supplementary material.

Supplementary file 1 (DOCX 32.0 KB)

## Data Availability

The data presented in this study are available on request from the corresponding author. The data are not publicly available due to the restriction of privacy and law.
