# Supplementary material for: Evaluation of extraction methods for co-isolation of nucleic acid from human saliva for forensic body fluid identification
Source: Int J Legal Med. 2025 Nov 7;140(2):667–83. doi: 10.1007/s00414-025-03641-9 (PMC12956961; doi:10.1007/s00414-025-03641-9)
Supplement: Supplementary file 1 — (DOCX 32.0 KB) [file 414_2025_3641_MOESM1_ESM.docx]

**Supplementary Information:**

*Table 1 Analysis of DNA quantity from five different saliva sample volumes (in triplicates) extracted using three different extraction kits by ND-1000. Significance differences are considered if P < 0.05 using Friedman test*

|  | DNA yield(ng/μL) | | | |
| --- | --- | --- | --- | --- |
| Extraction Method | Sample Volume (μL) | Median (ng/μL) | Range | P -value |
| Genomic DNA Kit | 400 | 47.70 | 5.80 | <0.0001 |
|  | 200 | 21.70 | 1.80 |  |
|  | 100 | 9.60 | 0.70 |  |
|  | 50 | 5.10 | 0.40 |  |
|  | 25 | 2.10 | 0.70 |  |
| QIAamp DNA Kit | 400 | 11.10 | 2.10 | <0.01 |
|  | 200 | 9.80 | 0.80 |  |
|  | 100 | 8.50 | 0.50 |  |
|  | 50 | 9.50 | 3.30 |  |
|  | 25 | 6.70 | 0.40 |  |
| miRNeasy Tissue Kit | 400 | 18.70 | 1.00 | <0.01 |
|  | 200 | 6.60 | 0.90 |  |
|  | 100 | 7.50 | 2.90 |  |
|  | 50 | 5.40 | 0.50 |  |
|  | 25 | 8.30 | 0.50 |  |

*Table 2 Analysis of RNA quantity from five different saliva sample volumes (in triplicates) extracted using three different extraction kits by ND-1000. Significance differences were considered if P < 0.05 using Friedman test*

|  | RNA yield (ng/μL) | | | |
| --- | --- | --- | --- | --- |
| Extraction Method | Sample Volume (μL) | Median (ng/μL) | Range | P-value |
| Genomic DNA Kit | 400 | 38.20 | 5.90 | <0.0001 |
|  | 200 | 19.20 | 1.30 |  |
|  | 100 | 9.30 | 0.10 |  |
|  | 50 | 3.90 | 0.10 |  |
|  | 25 | 1.50 | 0.50 |  |
| QIAamp DNA Kit | 400 | 7.90 | 0.70 | <0.001 |
|  | 200 | 5.70 | 1.10 |  |
|  | 100 | 4.90 | 0.40 |  |
|  | 50 | 4.80 | 0.90 |  |
|  | 25 | 4.30 | 1.70 |  |
| miRNeasy Tissue Kit | 400 | 15.10 | 0.50 | <0.0001 |
|  | 200 | 4.90 | 1.30 |  |
|  | 100 | 6.10 | 1.00 |  |
|  | 50 | 3.50 | 0.40 |  |
|  | 25 | 8.50 | 1.70 |  |

*Table 3 Analysis of miRNA quantity from five different saliva sample volumes (in triplicates) extracted using three different extraction kits by ND-1000. Significance differences were considered if P < 0.05 Friedman test*

|  | miRNA yield (ng/mL) | | | |
| --- | --- | --- | --- | --- |
| Extraction Method | Sample Volume (μL) | Median (ng/mL) | Range | P-value |
| Genomic DNA Kit | 400 | 701.00 | 61.00 | <0.0001 |
|  | 200 | 357.00 | 12.00 |  |
|  | 100 | 173.00 | 42.00 |  |
|  | 50 | 67.90 | 9.10 |  |
|  | 25 | 21.10 | 8.30 |  |
| QIAamp DNA Kit | 400 | 119.00 | 12.00 | 0.21 |
|  | 200 | 96.40 | 21.70 |  |
|  | 100 | 87.20 | 5.20 |  |
|  | 50 | 126.00 | 99.20 |  |
|  | 25 | 61.70 | 13.20 |  |
| miRNeasy Tissue Kit | 400 | 295.00 | 44.00 | <0.001 |
|  | 200 | 9.97 | 3.84 |  |
|  | 100 | 8.89 | 1.76 |  |
|  | 50 | 4.24 | 0.21 |  |
|  | 25 | 2.74 | 1.06 |  |

Table 4 Comparison of miRNA and reference gene expression level in saliva sample extracted from three different isolation kits

| Target | Genomic DNA Kit (Cq ± SD) | QIAamp DNA Kit (Cq ± SD) | miRNeasy Tissue Kit (Cq ± SD) |
| --- | --- | --- | --- |
| RNU44 | 33.35 ± 0.32 | 35.55 ± 0.84 | 33.83 ± 0.11 |
| RNU48 | 31.40 ± 0.36 | 35.07 ± 0.48 | 32.39 ± 0.08 |
| RNU6b | 35.33 ± 0.22 | 35.74 ± 0.22 | 35.33 ± 0.37 |
| miR-203 | 25.00 ± 0.02 | 26.18 ± 0.07 | 29.58 ± 0.01 |
| miR-205 | 26.78 ± 0.03 | 26.16 ± 0.08 | 26.80 ± 0.13 |

**Negative Controls:**

**NTC- undetermined**

**NRT-undetermined**
